# Pelvic organ prolapse and stress urinary incontinence: A review of etiological factors

**DOI:** 10.4103/0970-1591.32064

**Published:** 2007

**Authors:** Payal D. Patel, Kaytan V. Amrute, Gopal H. Badlani

**Affiliations:** University of Vermont College of Medicine, Burlington, VT, USA; * Department of Ob/Gyn, Woodhull Medical Center, Brooklyn, NY, USA; ** Department of Urology, Wake Forest University Baptist Medical Center, Winston-Salem, NC, USA

**Keywords:** Pathophysiology, pelvic organ prolapse, stress urinary incontinence

## Abstract

Although they may present with significant morbidity, pelvic organ prolapse and stress urinary incontinence are mainly afflictions that affect quality of life. To appropriately treat these entities, comprehension of the various theories of the pathophysiology is paramount. Utilizing a Medline search, this article reviews recent data concerning intrinsic (i.e., genetics, postmenopausal status) and extrinsic factors (i.e., previous hysterectomy, childbirth) leading to organ prolapse or stress incontinence.

Pelvic organ prolapse (POP) and stress urinary incontinence (SUI) are worldwide problems that affect the quality of life of millions of women. Although mortality is rare due to this health issue, it has been shown that self-perception of the body is significantly affected in those with symptoms.[1] Data from 1997 shows close to 350,000 pelvic organ prolapse operations performed in the United States alone.[2] Furthermore, it is expected that 11% of women over the age of 80 will undergo surgery for such conditions, with an additional 30% who will require a repeat surgery.[3] The direct cost based on this data is estimated to be over one billion dollars.[2]

The prevalence of urinary incontinence is thought to vary from 17-45% among adult women.[4] Likewise, 50% of parous women have pelvic organ prolapse.[3] The distribution of the severity of this condition among the general population has been studied since the development of a standardized evaluation method known as the pelvic organ prolapse quantification system (POP-Q). An initial study showed a bell-shaped curve distribution with the majority of women having Stage 1 or 2 prolapse in a population of 497 women greater than or equal to 18 years of age with a mean age of 44 years.[5] These stages were defined as the distal end of the prolapse being >1 cm and ≤ 1cm of the hymen during abdominal straining, respectively. A more progressive prolapse was noted in women greater than 40 years of age with 21% being in the age group of greater than the age of 70, suggesting that age plays a role. Although this study included close to 50% of African American and Caucasian women, it failed to stratify according to race.[5] A following study with a larger population of a comparable age group showed a similar curve to the previous study with the majority, again, having stage 1 and 2 prolapse. The difference among the studies, however, was the latter showing a greater prevalence of stage 3 and 4 prolapse (7% vs. 2-3%) which the authors hypothesized to be secondary to a more diverse population with an emphasis on Hispanic women.[6]

The pathophysiology of SUI and POP are related and can be considered multifactorial. These factors may be divided into intrinsic (genetics, age, postmenopausal status, ethnicity) and extrinsic (parity, history of previous hysterectomy, co-morbidities, occupation) components. Overall, irrespective of the inciting factor, the end result is the same: an anatomical defect in the endopelvic fascial layer leads to prolapse, often symptomatic.

## ANATOMY OF THE PELVIC FLOOR

### A brief overview

In normal anatomy, the suburethral endopelvic fascia plays a pivotal role in the prevention of SUI through attachments to the arcus tendineus laterally.[7] In addition, there are equal and opposite forces provided by the endopelvic ligaments and the components of the levator ani muscles that provide stability to the urethra.[8] Ulmsten suggested that the cause of incontinence is related to the difference in the equalizing forces of pressure on pelvic stabilizing components.[8] If an imbalance of these forces occurs, proper urethral closure is impeded. As an additive factor, there seems to be a weakened vaginal hammock which eliminates all support under the urethra.[8]

In regards to pelvic prolapse, the normal pelvic floor has several components that stabilize the pelvic organs in their respective positions. These include the levator ani muscle group, the sacral plexus and pudendal nerves and connective tissue which all act in conjunction to prevent prolapse through the genital hiatus. Innervated mainly by the pudenal nerve, the role of the muscle group is to lay the foundation upon which the pelvic organs rest. The connective tissue, which includes the endopelvic fascia and pelvic viscera, is responsible for attachment of the vagina on three levels: the proximal, mid-vagina and distal ends.[7] Proximal level I suspension indicates attachment of the upper two thirds of the vagina to the posterior pelvic wall via the uterosacral ligaments and paracolpium. The mid-vaginal section or level II, is secured through lateral attachments by the cardinal ligaments. The third level represents attachments of the distal vagina to marginal structures.[7]

In a healthy woman, an intact foundation is provided by pelvic floor muscles allowing for suspension of pelvic organs with little tension placed on the connective tissue. Subsequently, any impairment of this foundation leads to increased stretching and work of the suspensory ligaments and fascia of the pelvic organs. Although there is an effective malleable property of connective tissue to prevent prolapse even in the presence of damaged levator ani, there are a multitude of factors that work in concert for the development of a prolapse in certain women.[7,9]

## ETIOLOGY-INTRINSIC FACTORS

### Collagen matrix

**Figure 1:**
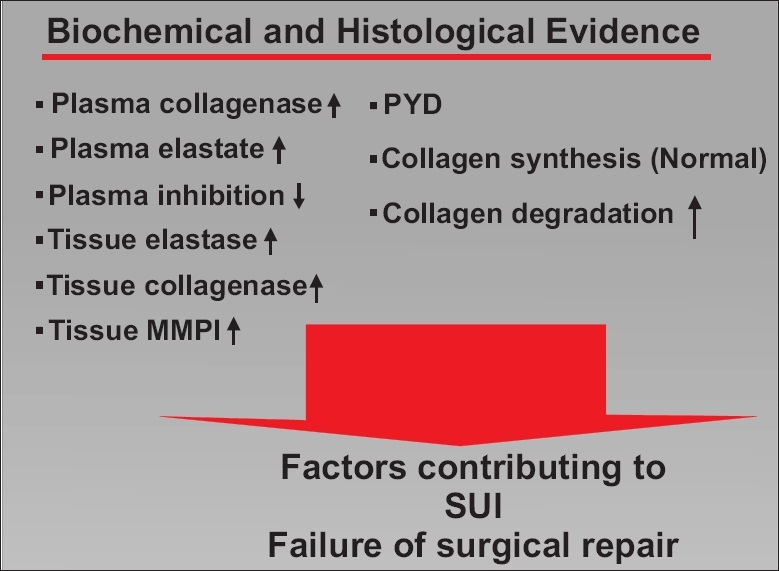
Factors contributing to abnormal collagen matrix

A presumed cause that is related to the failure of normal anatomy is an abnormal collagen matrix foundation, resulting in POP and SUI [Figure 1]. It has been well established that persons with connective tissue disorders such as Ehlers-Danlos and Marfans syndrome have a high rate of urinary incontinence and pelvic prolapse. The initial studies that were conducted in a female population with Ehlers-Danlos diagnosis showed a 20% higher incidence of SUI than those without any known connective tissue disorder.[10] Similarly, Carley *et al* confirmed this data in a population of women with either Ehlers-Danlos or Marfans syndrome. Interestingly, when comparing these two groups, their results showed a higher rate of prolapse in those with Ehlers-Danlos. Although the reason for this is not known, it was noted that the Ehlers-Danlos group had a higher parity rate.[11]

In women with stress incontinence or prolapse, Liapis *et al* compared paravaginal and uterosacral biopsies in women with POP with or without SUI and normal women.[12] In the group with both POP and SUI, a significant reduction of type III collagen was noted compared to the other groups.[12] An adjunct piece of evidence on the role of connective tissue lies in the study that observed greater frequency in abdominal hernias in those women with a prolapse, again suggesting the importance of functional collagen in the prevention of these disorders.[13] In further analysis of endopelvic fascia biopsies in women with incontinence, Chen *et al* concluded that this deficit is not due to a decreased production of collagen but due to increased degradation of nascent collagen.[14]

On a molecular level, collagenolytic activity was increased in women with stress incontinence as measured by a urinary collagen degradation product, helical peptide α1 (I) 620-633, when compared to a control group. After a 24h urine collection, the stress incontinent group had mean urinary concentrations of helical peptide α1 (I) 620-633 of 0.80 ± 0.13 μg/mg creatitine as opposed to 0.49 ± 0.06 μg/mg creatitine in the control group (*P* < 0.02).[15] Chen *et al* established similar conclusions by showing the up-regulation of matrix metalloproteinase mRNA by 80% (*P* = 0.05) which are responsible for collagen breakdown, as well as the suppression of its inhibitors by 34% (*P* =0.03) which act to regulate the collagen matrix. In conjunction, there was a 60% reduction per milligram of protein in the total collagen content in women with stress incontinence in comparison to control.[16]

Initial lessons garnered from individuals afflicted with hernias show that abnormal collagen metabolism may be a systemic event. Klinge *et al* demonstrated a significant decrease in collagen type I/III ratio in the skin of patients with indirect or direct hernia when compared to controls.[17] The authors concluded that hernias could be isolated manifestations of a systemic problem of collagen metabolism and this could delineate the high recurrence rates with repeated suture repair. Similar findings of systemic abnormal collagen metabolism have been noted in those with organ prolapse or stress incontinence. Gabriel *et al* recently preformed a histological and immunohistochemical comparison of uterosacral ligaments of patients with and without prolapse.[18] While there was no difference between collagen type I and smooth muscle content between the two groups, the prolapse group had a significantly higher expression of collagen type III (*P* < 0.001). Vaginal connective tissue in individuals with prolapse also demonstrates an increased expression of collagen type III fibers.[19] Analysis from our laboratory demonstrates reduced collagen content in pubocervical fascia and skin of women with stress incontinence compared to unaffected women.[14] Elastin, another component of connective tissue, is reduced secondary to increased elastolytic activity in the skin and endopelvic fascia of stress incontinent patients. Elevated proteolytic activity is evident in the plasma of patients with stress incontinence and provides further evidence of systemic process.[20]

### Genetics

In addition to mouse models, genetic involvement has also been measured *in vivo*. This has been researched via twin and family populations. Analysis of a group of women with a mean age of 44 years with stage 3-4 prolapse demonstrated a five-fold higher occurrence in members of their family. Inheritance in these families was of the dominant type with a high penetrance.[21] A twin study was conducted, to show a genetic component in these conditions, on a population of nulliparous women, aged 18-24 years, to demonstrate inheritance of bladder neck mobility as compared to nontwin and dizygotic sisters. Although there was limited power secondary to a small patient population, the results indicated a 59% genetic variability leading to a similar conclusion of positive genetic involvement with heritability being possible.[22]

### Aging

In addition to genetics, age has been recognized as an intrinsic factor in the development of urinary incontinence and pelvic prolapse. It is thought that advancement in age is associated with a higher rate of pelvic dysfunction. This is hypothesized to be secondary to a multitude of factors including the fall in estrogen during the postmenopausal period, as well as normal physiologic advancement of the pelvic floor components. Swift *et al* supported this theory by showing an increase in the odds ratio for pelvic prolapse from 1.04 to 1.46 for a change in 10 years of age.[6] An interesting study which concluded contrary findings, however, determined no risk associated with age in regards to prolapse.[23] The authors went even further to state that advancement in age led to a lower prolapse rate. Using the definition of a prolapse being the leading edge ≥ 0 cm, women were stratified into four age groups: ≤ 63, 64-68, 69-72 and >72 years of age. The number of prolapse were 25, 41.2, 20.6 and 13.2, respectively (*P* =0.04). This study, however, has several limitations with the most prominent being the lack of the POPQ examination method as the best means to define a prolapse. In essence, these results may have been underestimated.[23]

### Ethnicity

The final intrinsic factor involved in these disorders is ethnicity. A study analyzing a comparable group of African American and Caucasian women with a mean age of 57 and mean parity of 2.6, found racial differences in the areas of urethral closure pressures, cystometric capacity and the number of patients affected by SUI and detrusor instability. The African American group had greater maximum pressure with urethral closure and a lower maximal cystometric capacity in those who displayed urinary incontinence. Race was also a predictor of both detrusor instability and stress incontinence with a higher rate being observed in the African American population. As significant as these findings are, the major factor neglected in this study was the mode of delivery in each group which may have influenced the results obtained.[24] The first study to research Asian women *in vivo* revealed the difference that exists in pelvic floor strengths between nulliparous women in comparison to Caucasians. Asian women had thicker pelvic floor ligaments and fascia whereby preventing prolapse from readily occurring.[25]

Further studies performed by Duong *et al* in comparing the four ethnicities of African American, Caucasian, Hispanic and Asian women found similar results to previous studies. It was noted, however, that the Asian population had higher maximal cystometric capacity (446 ml ± 125) with lower maximal urethral closure pressure (48 cmH_2_ O ± 22) when compared to the African American women (402 ml ± 117, 58 ± 23) making them comparable to the Caucasian and Hispanic populations. Also of significance, Hispanic women had the highest rate of stress incontinence (67%) in this comparison, with African Americans (42%) having the lowest. Detrusor instability data exhibited the contrary association. Caucasian and Asians proved to be fairly comparable in regards to these conditions with 15% of Caucasians and 14% Asians affected. It should be considered, however, that the mean age in the Asian group was seven years younger than that of the African Americans creating the possibility of an even more dramatic disparity between these two ethnicities.[26]

## ETIOLOGY - EXTRINSIC FACTORS

### Parity

Of extrinsic factors promoting pelvic organ prolapse and stress incontinence, childbirth seems to have the biggest effect. To date the largest study to identify this correlation was the Women's Health Initiative. With a population size close to 28,000 postmenopausal women, the presence of a prolapse was determined. Data analysis showed a significant correlation of prolapse with childbirth, with a cystocele being the most frequent to develop. Furthermore, the most significant factor was the initial childbirth, with each additional birth bringing the odds ratio to 1.1-1.21, depending on the type of prolapse.[27] Patel *et al* demonstrated a similar trend in their analysis of the effect of parity on urinary incontinence while limiting the bias of the data by including only vaginal births. Their results showed an odds ratio of 2.3 with parity of one and 2.8 with an additional birth. In regards to POP, analysis of previous data calculated an odds ratio of 4.0 and 8.4 with parity one and two, respectively, without differentiating between different modes of delivery.[28]

Many have speculated the protective nature of Caesarian delivery in terms of pelvic prolapse since it limits the damage done to the pudendal nerve and the levator ani muscles. This was tested by Sze *et al* in a comparison between the two modes of delivery in a patient population of 94 nulliparous women of mean age 22.1 years. Of these, 50% had spontaneous vaginal delivery, 30% underwent Caesarean births and 20% underwent operative vaginal birth. Prolapse evaluation was conducted at 36 weeks antepartum and six weeks postpartum. During the postpartum phase, the Caesarean group surprisingly had a slightly greater rate of new pelvic prolapse of approximately 3%. However, a greater number of women in the spontaneous vaginal delivery population had a more severe prolapse with a difference amounting to 9%. Finally, no difference was noted on a two-stage severity scale between the groups. Of interesting note is that this study found most of the women in both groups to have an anterior defect predominantly as seen in many other prolapse studies. Although these findings were the first of its kind, only limited extrapolations may be made due to the small population size and lack of baseline assessment prior to the 36-week antepartum time period.[29]

Contrary to previous studies, Kukacz *et al* demonstrated the protective effect of Caesarean delivery in the development of POP and SUI. Using a standardized questionnaire, 4,458 women were assessed with odds ratios calculated. The rates of POP in nulliparous, Caesarean delivery and vaginally parous women were 4%, 4% and 8% (*P* < 0.05), respectively. The rates of SUI in the same populations were 8%, 11% and 18% (*P* < 0.05), respectively. Furthermore, the rates of POP in the unlabored Caesarean delivery and the labored Caesarean delivery were 1% and 7% (*P* =0.043), respectively. The same trend was observed in the same populations with SUI with the unlabored group having a prevalence of 5% and labored having 13% (*P* =0.065) where labored was not defined in time or obstetrical terms. Further analysis of numbers needed to treat showed that seven women needed to deliver by Caesarean only or five women needed to deliver by unlabored Caesarean specifically to prevent one pelvic floor disorder.[30]

The mechanism of action behind the effect of childbirth leading to a prolapse was tested in a novel prospective study conducted by Sultan *et al*. Consistent with the effects of parity as stated above, pudendal nerve terminal motor latency measurements proved the greatest effect on prolapse from the first vaginal delivery. This was shown to be due to perineal descent at the time of labor. Also in concert with previous findings, this study did not find a difference in the different modes of delivery. In fact, pudendal damage was seen in women who had labored for ≥ 20h with fetal head engagement followed by a Caesarean delivery.[31]

### Hysterectomy

The effect of hysterectomy on POP and urinary incontinence has long been evaluated. The disruption of endopelvic fascia, uterosacral-cardinal ligament supports and local nerve supply, by hysterectomy can conceivably impair the pelvic floor. Previous debate centered on the route of hysterectomy (i.e., vaginal versus abdominal) and, in abdominal cases, benefits of preservation of the cervix (i.e., subtotal hysterectomy). Retention of the cervix during hysterectomy prevents the disruption of the uterosacral and cardinal ligaments thereby preventing possible future prolapse. Older studies, however, have been observational and limited by retrospective data collection.[32]

A randomized, double-blind trial by Thakar *et al* compared the outcomes of total hysterectomy and subtotal hysterectomy with a one-year follow-up.[33] The preoperative and postoperative rates of urinary frequency, stress incontinence, urgency, poor stream and incomplete bladder emptying did not differ significantly between the two groups. Urodynamic studies showed a reduction in stress incontinence after surgery in both groups.[33] Also, at one-year follow-up, two patients (1.5%) in the subtotal hysterectomy group presented with cervical prolapse.

Another randomized, controlled, multi-center trial (n=276) using questionnaires in Denmark revealed that a significantly (*P* =0.043) smaller proportion of women complained of urinary incontinence after total abdominal hysterectomy compared to subtotal hysterectomy (9% vs. 18% or 2.08, 95% CI 1.01-4.29) after one year follow-up.[34] Furthermore, a small number of patients (n=2) reported cervical stump prolapse in the subtotal hysterectomy group (n=140) and, when compared to the total hysterectomy group, the differences were statistically insignificant.

A recent analysis of short-term outcomes, such as hospital duration, return to normal activities and number of febrile episodes, showed greater overall benefits with vaginal hysterectomy compared to abdominal hysterectomy.[35] However, randomized trials are necessary to evaluate long-term outcomes of pelvic prolapse and incontinence as current data is lacking comparing the two modalities.[35]

### Hormone replacement therapy

The presence of estrogen receptors throughout the urogenital tract implies that estrogen may have physiologic effects on the continence mechanism. Estrogen increases urethral blood flow, α-adrenergic receptor sensitivity, urethral closure pressure and improves cellular maturation in the bladder, trigone and urethra.[36] These findings are the basis for hormone replacement therapy (HRT) in clinical practice for treatment of incontinence. While earlier studies have shown conflicting results, current research demonstrates increased risk of incontinence with HRT use.

One of the initial studies conducted by Fantl *et al* looked at women with measured low serum estradiol and a diagnosis of stress incontinence based on urodynamics. The results of this small population showed no effect of 0.625 mg of estrogen for 30 days followed by 10 mg of medroxyprogesterone for 10 days over a three-month period.[37] The heart and estrogen/progestin replacement study (HERS) was a randomized, placebo-controlled, double-blind trial designed to evaluate daily oral conjugated estrogen (0.625 mg) with medroxyprogesterone acetate (2.5 mg) for the prevention of cardiac events in women with established disease.[36] After four years of treatment, the study noted that oral estrogen and progestin caused urge and stress urinary incontinence. In the large sample population, 64% of women in the hormone replacement group reported weekly incontinence versus 49% in the placebo group (*P* < 0.001). Patients on hormone therapy had 50% higher odds of urge incontinence and 70% higher odds of stress incontinence.[36]

The nurses health study, a large prospective cohort study, noted a significant increase in the risk of developing incontinence with the use of estrogen and progestin therapy.[37] The risk was similar for users of estrogen alone and estrogen with progestin, along with users of oral or transdermal estrogen. Hendrix *et al*, using participants in the women's health initiative (WHI), ascertained as well that users of estrogen alone or estrogen with progestin developed a higher risk of urinary incontinence and HRT should not be utilized to treat or prevent incontinence.[38]

Estrogen may cause these adverse effects through profound effects on collagen metabolism, i.e., stimulation of collagen degradation by increased matrix metalloproteinase-2 activity.[36,38,39] Weakened anatomical support with increased vesicular pressure can promote incontinence. At our institution, we demonstrate similar findings: elastolytic activity was unchanged in the collagen matrix of endopelvic fascia fibroblasts from women with SUI (n=8), but was greater than five-fold elevated in the collagen matrix of endopelvic fascia fibroblasts from women without SUI (n=6) after a one-day exposure to 17-β-estradiol and remained elevated over the three days of treatment.[40] This is consistent with the observation that menopausal HRT increases the risk of incontinence in continent women. Differences in the expression of ERs and elastolytic activity suggest that local tissue may have differential sensitivity and/or responsiveness to estrogen.[40] A similar consequence of estrogen administration may occur with POP. Liu *et al* analyzed the proliferation of fibroblasts derived from cardinal ligaments of patients with and without prolapse after 17β-estradiol administration.[41] The fibroblasts from the prolapse group showed a significantly lower proliferative rate than that of the control group at all estradiol concentrations. Clinically, HRT may not be beneficial in prolapse treatment.[41]

A recent study measuring the effects of oral versus topical estrogen treatments in a postmenopausal population of women with a history of hysterectomy showed opposite results to these previous studies.[42] The authors demonstrated a decrease in lower urinary track symptoms such as frequency and nocturia after a three-month treatment period. Oral treatment resulted in a reduction in frequency by 51.9% (*P* < 0.01) and in nocturia by 22.3% (*P* < 0.05), whereas topical cream reduced frequency and nocturia by 33.3% with *P* < 0.05 and *P* < 0.01, respectively. Subjectively, SUI improved by 72.7% secondary to oral treatment and by 60% after topical treatment, although there was no statistical significance seen via objective measures.[42] To support this from a histological perspective, Lang *et al* noted a direct correlation between the estrogen receptor value and years of postmenopausal status in those with SUI and POP suggesting the potential benefit of estrogen therapy.[43]

### Co-morbidities

Damage to the components of the pelvic floor can occur through chronic transmitted intraabdominal downward pressure. One mechanism is through chronic cough secondary to a chronic pulmonary disease which has been tested by Rinne *et al* that proved asthma to be a greater risk factor of approximately 12%.[13] Another study mimicked these effects with an approximate 11% risk in the development of urinary incontinence.^3^ In correlation with this, a large cross-sectional study found an increase of 56% in the prevalence rate among patients who currently smoke.[44]

Obesity also seems to impact pelvic floor function. The WHI found patients with a BMI in the range of 25-30 kg/m^2^ to have 31% of uterine prolapse, 38% of rectocele and 39% of cystocele. These percentages increased with increased BMI values.[27] Richter *et al* calculated a gradual rise of 3% in incontinence with every 1 unit increase in BMI. It should be noted, however, that incontinence in this study was measured via a bladder diary versus urodynamics.[44]

### Occupation

Although there is little data on the risk of occupation on POP and stress incontinence, available studies seem to be consistent with the idea of profession impacting pelvic functionality. An initial Danish study concluded an occupation that requires heavy lifting to be a potential cause of prolapse as manifested in a greater number of prolapse surgeries in this population.[45] In a study by Chiaffarino *et al*, housewives were significantly at a higher risk of prolapse than women in managerial positions under the assumption that they encounter greater physical labor.[46] To further stratify different occupations with their concurrent risks of prolapse, Woodman *et al* found the greatest odds ratio of 7.75 in factory workers and laborers. This was followed subsequently by housewives, service workers, technical workers and professionals.[47] A socioeconomic assessment using an occupation score which took into account education and income, concluded that there was a 4% decrease in the prevalence of incontinence with a calculated 10 unit increase in the occupation score. As speculated, this increase correlates to a higher education and income, without considering the extent of labor.[44]

## CONCLUSION

Pelvic organ prolapse and stress urinary incontinence are common health problems affecting women of all age groups worldwide. There are many factors that contribute to the development of these conditions such as collagen integrity, genetics and ethnicity, age, parity, occupation and co-morbid health issues. Certainly, a clear understanding of the etiology is important in the assessment and diagnosis of patients. Although there is significant data currently available, there is an obvious need for long-term, large, prospective research in the area of the pathophysiology of these conditions in order to discover novel therapies, as well as focus on the preventing factors.
